# Multi-omic profiling reveals the impact of keratinase kerZJ on mouse gut homeostasis

**DOI:** 10.71150/jm.2509011

**Published:** 2025-12-31

**Authors:** Xueqing Gan, Yijiao Wen, Si Chen, Famin Ke, Siyuan Liu, Zening Wang, Chunhua Zhang, Xuanting Wang, Qin Wang, Xiaowei Gao

**Affiliations:** 1 Department of Cardiovascular Medicine, Xuyong People's Hospital, Luzhou 646400, P. R. China; 2 Green Pharmaceutical Technology Key Laboratory of Luzhou, School of Pharmacy, Southwest Medical University, Luzhou 646000, P. R. China; 3 Dazhou Vocational College of Chinese Medicine, Dazhou 635000, P. R. China; 4 State Key Laboratory of Neurology and Oncology Drug Development, Nanjing 210000, P. R. China

**Keywords:** keratinase, gut microbiota, proteome, transcriptome, feed additive

## Abstract

Keratinase kerZJ is a multifunctional protease with potential as a feed additive and functional ingredient. Here we performed an integrated multi‑omics evaluation of its biosafety and impact on gut homeostasis in mice. Our findings confirm that kerZJ is well-tolerated, with no evidence of systemic toxicity or intestinal epithelial damage. Integrated transcriptomic and proteomic analyses revealed that kerZJ reinforces intestinal barrier integrity by upregulating extracellular matrix components, including collagen IV, and modulates mucosal immunity by enhancing B-cell activation and antimicrobial peptide defenses without inducing inflammation. Furthermore, kerZJ administration led to a significant upregulation of digestive enzymes and a dose-dependent increase in short-chain fatty acids production. Microbiome analysis showed that while high-dose kerZJ altered community composition, it enriched for beneficial taxa like *Lactobacillaceae* and did not induce dysbiosis. These results demonstrate that kerZJ safely enhances gut barrier function, promotes a favorable immune and metabolic environment, and fosters a resilient gut ecosystem, supporting its development as a safe feed additive and nutraceutical component.

## Introduction

Keratinases are a specialized class of proteases capable of hydrolyzing keratin, a highly stable fibrous protein rich in disulfide bonds, hydrogen bonds, and hydrophobic interactions, which renders it resistant to degradation by common proteases such as pepsin and trypsin (Pan et al., 2021). These enzymes are primarily produced by keratinolytic microorganisms, including bacteria, actinomycetes, and fungi, often isolated from environments abundant in keratinous wastes such as feathers, hair, and wool (Vidmar and Vodovnik, 2018). Keratinases are classified mainly into two families: subtilisin-like serine proteases (S8A) and thermolysin-like metalloproteases (M4), based on their catalytic mechanisms and structural features (Hassan et al., 2020). Over the past few decades, research on keratinases has advanced significantly, driven by their catalytic versatility, with studies focusing on enzyme isolation, recombinant expression, biochemical characterization, and protein engineering to enhance stability, activity, and substrate specificity (Pei et al., 2025; Yan et al., 2024). These developments have expanded their potential from waste management to biomedical applications, highlighting their role in sustainable biotechnology.

In the feed industry, keratinases serve as valuable additives that convert keratin-rich agricultural waste into digestible proteins and bioactive peptides, thereby improving nutrient utilization and reducing environmental pollution (Zhang et al., 2023). Studies have demonstrated that dietary supplementation with keratinases enhances animal growth performance, increases the digestibility of feather meal, and promotes the absorption of essential amino acids (Odetallah et al., 2005; Zheng et al., 2023). Furthermore, keratinases have shown promising applications in leather processing, detergent formulation, and cosmetics production (Gupta et al., 2013; Li, 2021). Recent investigations have revealed their potential in biomedical fields, including the treatment of dermatological conditions such as psoriasis and acne, and enhancement of drug delivery through the stratum corneum (Thadiyan et al., 2025).

KerZJ, a versatile keratinase recently identified by our group from *Stenotrophomonas* sp. LMY, exhibits remarkable multifunctional properties that distinguish it from conventional keratinases (Peng et al., 2023). This enzyme demonstrates keratin-degrading ability under mild conditions, efficiently converting feather waste into valuable protein hydrolysates that can be utilized as feed supplements. Importantly, kerZJ possesses the capability to hydrolyze scrapie prion proteins (PrPSc), the causative agents of fatal neurodegenerative transmissible spongiform encephalopathies, suggesting its potential application in preventing prion-related diseases in livestock (Peng et al., 2023; Zhu and Aguzzi, 2021). Additionally, kerZJ exhibits significant fibrinolytic activity both in vitro and in vivo, demonstrating therapeutic efficacy in murine thrombosis models without adverse effects (Peng et al., 2023; Sharma et al., 2021). This multifunctional profile positions kerZJ as a promising candidate for development as both a feed additive to enhance protein utilization and prevent prion infections, and as an active ingredient in functional food products with thrombolytic properties, complementing existing nattokinase-based nutraceuticals in the prevention and management of cardiovascular diseases (Weng et al., 2017).

The present study employs an integrated multi-omics approach to comprehensively evaluate the impact of dietary kerZJ supplementation on mice gut homeostasis and to assess its biosafety profile. Through transcriptomic and proteomic analysis, we characterized the host gene expression profiles in intestinal tissues, revealing the molecular pathways modulated by kerZJ administration. Remarkable alterations in digestive enzymes, extracellular matrix proteins, and immune mediators were detected. Microbiome analysis demonstrated the effects of kerZJ on gut microbial community structure and diversity, with particular emphasis on beneficial bacterial populations and metabolic functions. Our results indicate that kerZJ supplementation reinforces basal lamina and mucosal structural support, modulates immune responses favorably, and promotes a balanced gut microbiota composition without inducing pathological changes or dysbiosis. This comprehensive biosafety assessment provides crucial evidence supporting the potential application of kerZJ as a safe and effective feed additive and functional food component, laying the foundation for its further development and commercialization in animal nutrition and human health applications.

## Materials and Methods

### Materials

The ELISA kit for secretory IgA (sIgA) and biochemical assay kits for total bilirubin, malondialdehyde (MDA), creatinine, superoxide dismutase (SOD), alanine aminotransferase (ALT) activity, and glutathione (GSH) were purchased from Elabscience Co., Ltd. (China) or Jiancheng Bio-Engineering Institute Co., Ltd. (China). The E.Z.N.A. Stool DNA Kit was obtained from Omega Bio-tek (USA). Antibodies against zonula occludens-1 (ZO-1), claudin-1, and occludin, as well as FITC- or Cy3-conjugated secondary antibodies, were sourced from Servicebio Co., Ltd. (China) or Proteintech Group Co., Ltd. (USA). Standards for short-chain fatty acids (SCFAs) were purchased from Sigma-Aldrich (USA). All other chemicals were of analytical grade and obtained from commercial suppliers.

### Animal and feeding experiments

Male C57BL/6J mice (5 weeks old) were obtained from Chongqing Tengxin Biotechnology Co., Ltd. (China) and maintained under specific pathogen-free conditions on a 12‑h light/dark cycle, with ad libitum access to food and water. Following a one-week acclimation period, mice were randomly allocated into three groups (n = 8/group). The control group (Ctrl) received 100 μl of phosphate-buffered saline (PBS) daily via oral gavage. Concurrently, the treatment groups were administered either a low dose (KerL; 100 FU/kg) or a high dose (KerH; 1000 FU/kg) of kerZJ, prepared in the same vehicle and administered daily in an identical manner. At the conclusion of the 30-day experiment, fresh fecal samples were collected from each animal into sterile tubes for gut microbiota analysis. Subsequently, the mice were euthanized by anesthetic overdose, and serum and major organs were harvested for subsequent biochemical and histopathological analyses (Wu et al., 2023; Zhang et al., 2023).

### Hematoxylin and eosin staining

For histopathological analysis, tissue samples were fixed in 10% neutral buffered formalin and subsequently embedded in paraffin. The resulting blocks were sectioned at 4 μm, stained with hematoxylin and eosin (H&E), and then examined for morphological changes (Gao et al., 2025; Liang et al., 2023). Representative images were captured using a digital microscope (BA210, Motic, China).

### ELISA and biochemical assays

The levels of sIgA, total bilirubin, MDA, creatinine, and GSH, as well as the activities of SOD and ALT, were determined using commercial kits according to the manufacturer’s instructions (Liang et al., 2023).

### Immunofluorescence staining and evaluation

Immunofluorescence analysis was performed to assess the expression and localization of the tight junction proteins ZO-1, claudin-1, and occludin in intestinal tissue (Gao et al., 2025). Briefly, tissue sections were fixed in paraformaldehyde, washed with ethanol, and subjected to antigen retrieval. Following blocking with bovine serum albumin (BSA), the sections were incubated overnight at 4°C with primary antibodies specific for ZO-1, claudin-1, and occludin. Subsequently, the sections were treated with FITC- or Cy3-conjugated secondary antibodies and visualized under a fluorescence microscope (Nikon, Japan).

### Transcriptomic analysis

Total RNA was extracted from intestinal tissue samples using the MJZol Total RNA Extraction Kit (Majorbio, China) following the manufacturer’s protocol (Yi et al., 2021). The concentration and purity of the extracted RNA were quantified using NanoDrop 2000 spectrophotometer, and RNA integrity was verified by 1% agarose gel electrophoresis. Subsequently, mRNAs were enriched from the total RNA using oligo(dT)-conjugated magnetic beads and then fragmented into lengths of approximately 300 bp. Strand-specific cDNA libraries were constructed using the TruSeq Stranded Total RNA Library Prep Kit (Illumina, USA) and sequenced on an Illumina HiSeq platform. Raw sequence reads were filtered to remove low-quality data using the fastp software (Yi et al., 2021). Downstream bioinformatics analyses were performed on the Majorbio Cloud Platform (https://www.majorbio.com).

### Proteomics analysis

For proteomic analysis, proteins were extracted from intestinal tissue samples via homogenization and non-contact cryogenic sonication in a lysis buffer supplemented with a protease inhibitor cocktail. Protein concentration was determined using the bicinchoninic acid (BCA) assay. For each sample, a 100 μg aliquot of protein was subjected to tryptic digestion. The resulting peptides were subsequently analyzed by a Vanquish Neo UHPLC system coupled to an Orbitrap Astral mass spectrometer (Thermo Fisher Scientific, USA). The raw data files were processed using Spectronaut software for protein identification and quantification, presumably employing a data-independent acquisition (DIA) workflow (Yi et al., 2021). Downstream bioinformatics analyses were conducted on the Majorbio Cloud Platform (https://www.majorbio.com).

### Microbiome analysis

For gut microbiome analysis, bacterial genomic DNA was extracted from fecal samples, and the bacterial 16S rRNA gene were amplified by PCR using the extracted DNA as template. Amplicon libraries were constructed from the PCR products and sequenced on an Illumina MiSeq platform. Raw sequence reads were quality-filtered using Trimmomatic and taxonomically annotated against the SILVA ribosomal RNA gene database. Microbial community diversity was analyzed and compared using the Mothur and QIIME 2 software packages. Linear discriminant analysis effect size (LEfSe) was employed to identify bacterial taxa with significantly differential abundance between groups at the phylum-to-genus levels (LDA score > 2, P < 0.05). Additional bioinformatics analyses were performed as previously described with the Majorbio Cloud Platform (Ke et al., 2024).

### SCFAs determination

The quantification of SCFAs was achieved using a targeted liquid chromatography-tandem mass spectrometry (LC-MS/MS) method. For sample preparation, 50 μl of serum was homogenized with 100 μl of an acetonitrile-based solution, followed by ultrasonication for 30 min at 5°C. Cell debris was pelleted by centrifugation at 13,000 × g for 15 min at 4°C. The resulting supernatant was then subjected to chemical derivatization by adding 20 μl of 200 mM 3-nitrophenylhydrazine hydrochloride (3-NPH·HCl) and 20 μl of 120 mM N-(3-dimethylaminopropyl)-N'-ethylcarbodiimide hydrochloride containing 6% pyridine. Prior to analysis, the mixture was diluted to 750 μl with a 50% aqueous acetonitrile solution and analyzed on a UHPLC-Qtrap system. Peak integration and compound quantification were performed using AB Sciex Analyst^®^ software, with all automated results manually verified for accuracy.

### Statistical analysis

Data were analyzed in GraphPad Prism (v8.0) and are presented as the Mean ± SD. Group comparisons were performed using either an unpaired Student's t-test (for two groups) or a one-way ANOVA with Tukey's post-test (for multiple groups). A result was deemed statistically significant if the p value was less than 0.05.

## Results

### Evaluation of the biosafety of kerZJ

**Fig. 1.**
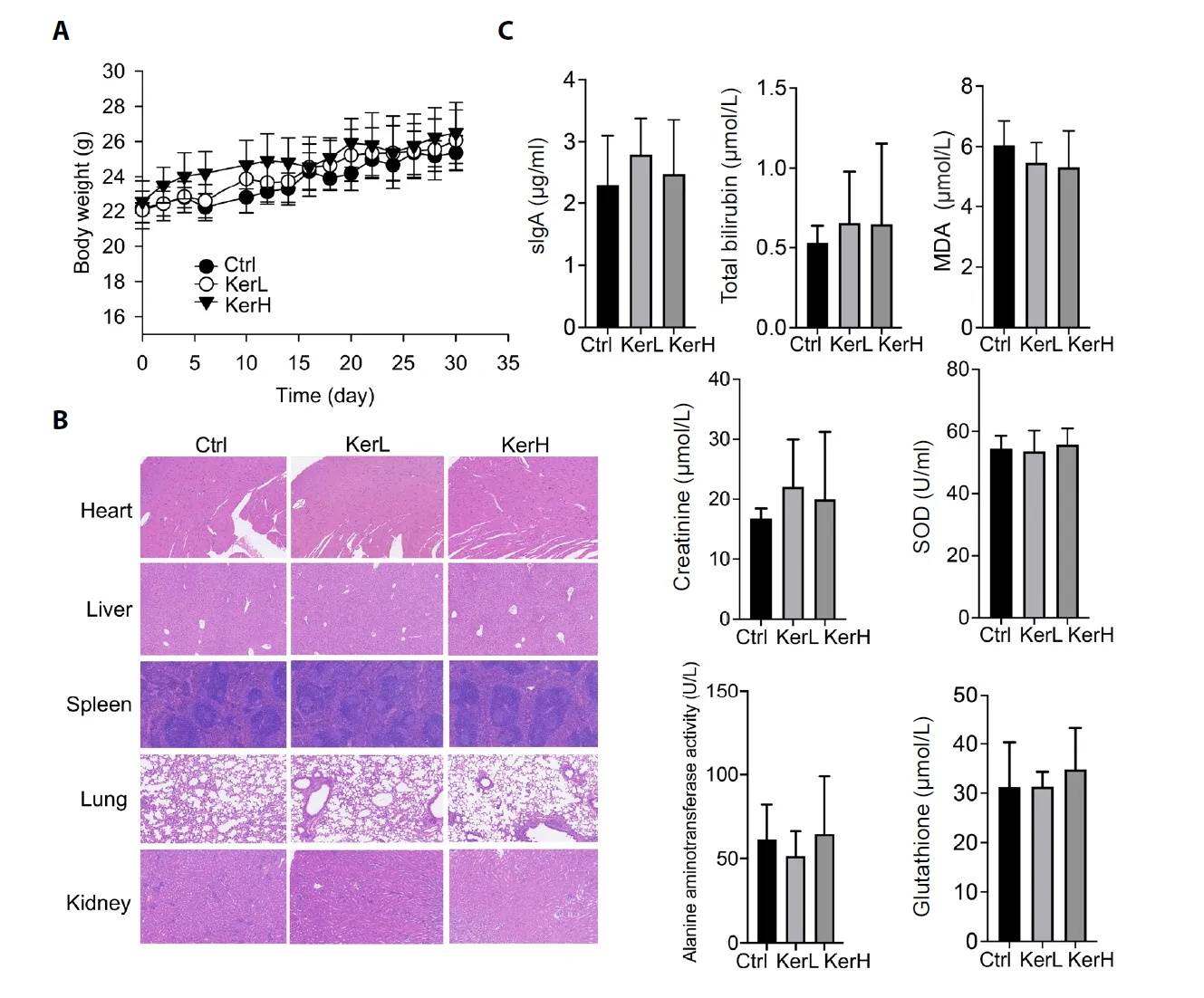
Evaluation of the physiological toxicity of kerZJ in mice. (A) Effects of kerZJ on body weight gain. (B) H&E staining of major organs across the three groups. (C) Serum biochemical parameters following kerZJ administration. Data are presented as Mean ± standard deviation (SD).

The biosafety of kerZJ was evaluated in mice. Following administration, no significant differences in the final body weights were recorded among the three experimental groups, despite a transient and slight elevation in the KerH group during the first week (Fig. 1A). The toxicity of kerZJ was further assessed by comparing pathological changes in major organs and serum biochemical factors. As shown in Fig. 1B, no obvious pathological abnormalities were observed in the major organs of mice in the kerZJ-treated groups. Additionally, serum levels of sIgA, total bilirubin, malondialdehyde (MDA), creatinine, superoxide dismutase (SOD), alanine aminotransferase (ALT) activity, and glutathione (GSH) in kerZJ-administered mice were comparable to those of the control mice (Fig. 1C). These findings indicate that kerZJ exhibits no systemic physiological toxicity in mice following oral administration. Given that kerZJ directly interacts with the gastrointestinal tract upon ingestion, subsequent analyses focused on its effects on gut homeostasis in mice.

### Effects of kerZJ on intestinal epithelial tight junction proteins in mice

**Fig. 2.**
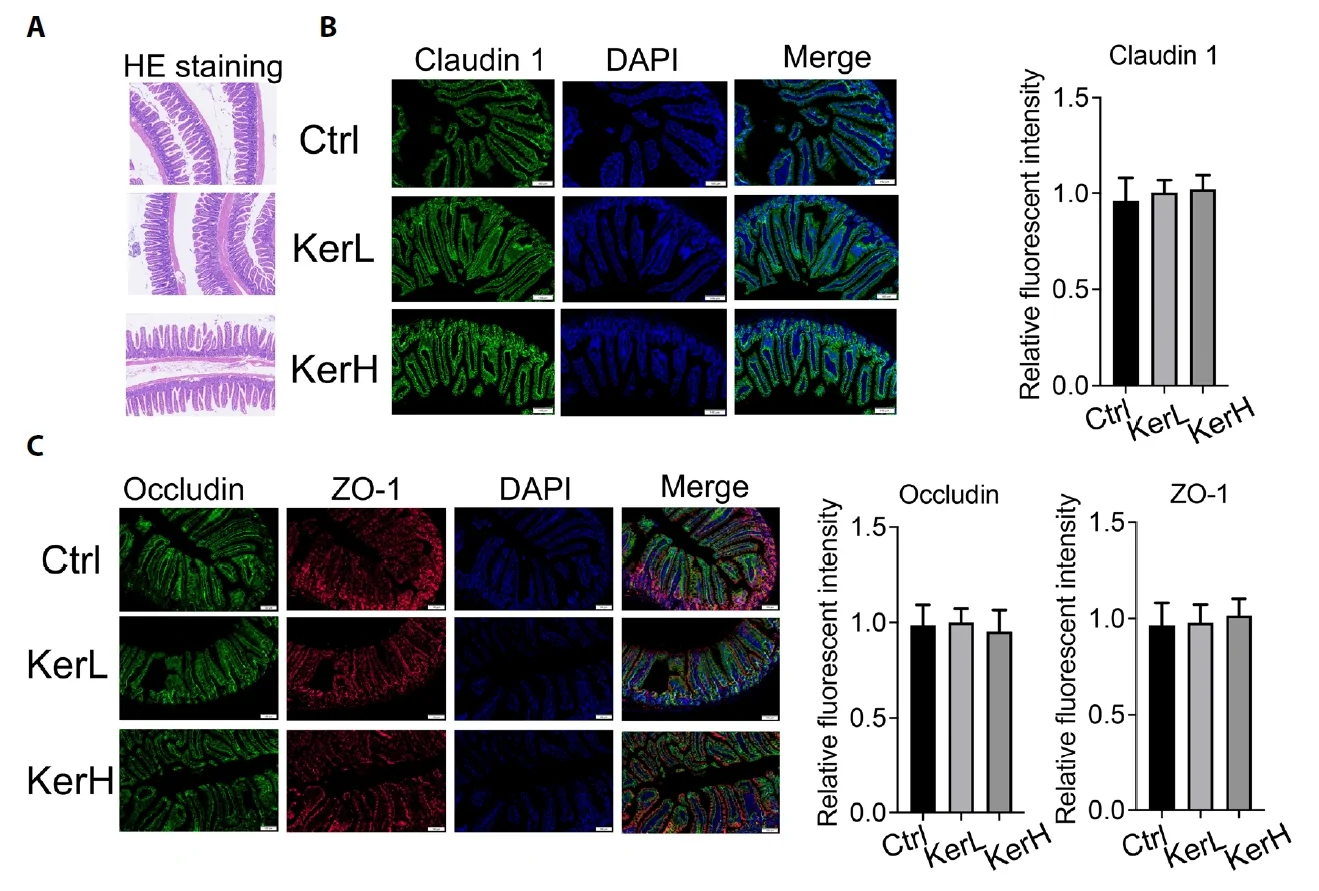
Expression of intestinal epithelial tight junction proteins in mice. (A) H&E staining of intestinal tissues. (B) Immunofluorescence staining with anti-claudin-1 antibody; the bar plot (right) shows the relative fluorescence intensity of claudin-1 across the three groups. (C) Immunofluorescence staining with anti-ZO-1 and anti-occludin antibodies; the bar plot (right) shows the relative fluorescence intensities of ZO-1 and occludin across the three groups. Data are presented as Mean ± standard deviation (SD).

Based on H&E staining, the intestinal morphology of mice in the KerL and KerH groups showed no discernible differences compared with the Ctrl group (Fig. 2A). To assess whether KerZJ adversely affects epithelial barrier integrity, we performed immunofluorescence analyses to quantify the tight junction proteins claudin‑1, ZO‑1, and occludin. As shown in Fig. 2B, claudin‑1 fluorescence intensity was comparable across all groups, and similar results were observed for ZO‑1 and occludin (Fig. 2C). Together, these data indicate that KerZJ does not significantly affect intestinal epithelial tight junction proteins in mice.

### Effects of kerZJ on the intestinal transcriptome in mice

**Fig. 3.**
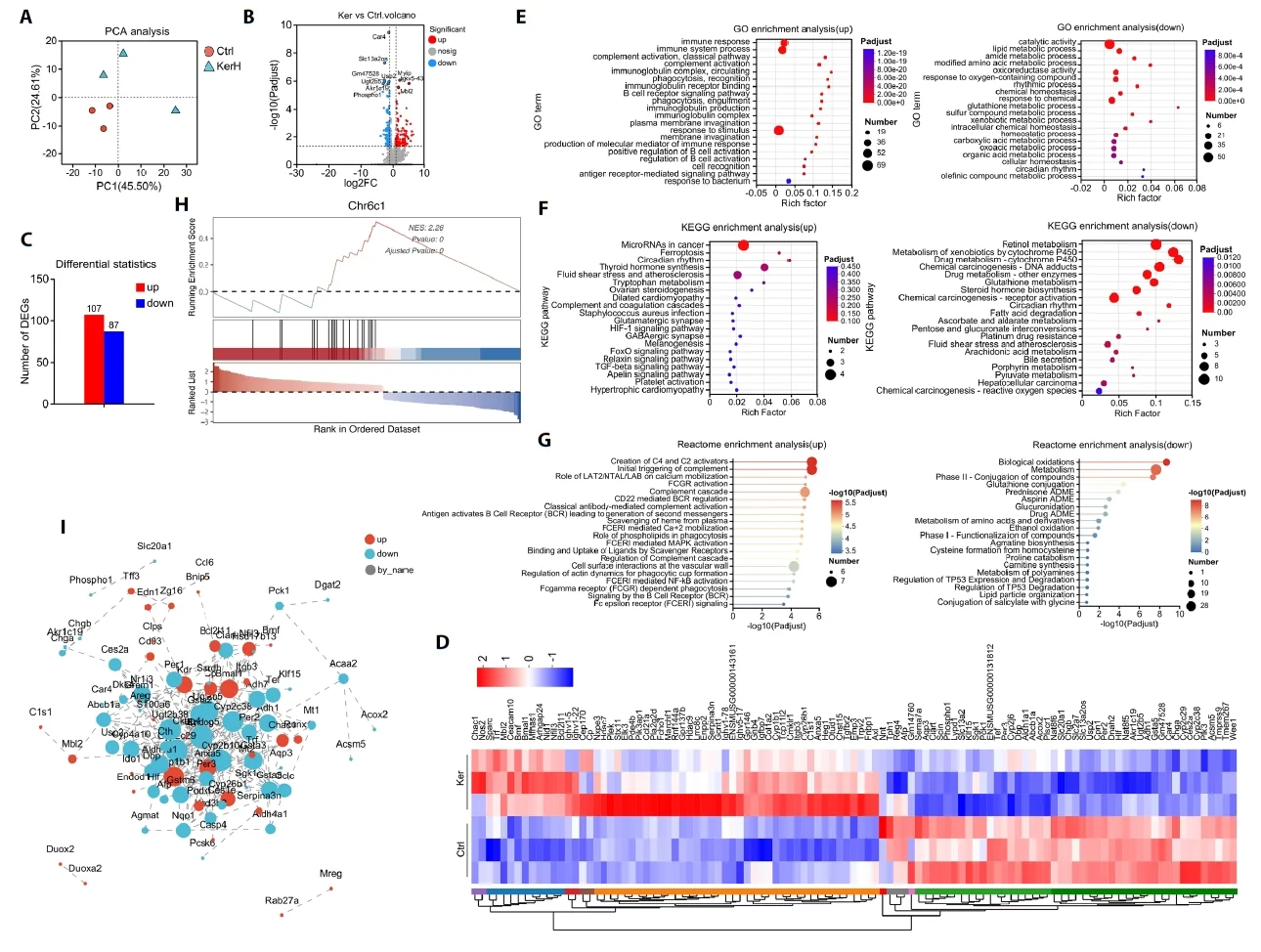
Transcriptomic alterations and functional implications in mice. (A) Principal component analysis (PCA) of transcriptomic profiles across samples. (B) Volcano plot showing significantly differentially expressed genes. (C) Bar chart indicating the numbers of up- and down-regulated genes. (D) Heatmap of differentially expressed genes across samples. (E) Gene Ontology (GO) enrichment of up- and down-regulated genes. (F) KEGG pathway enrichment of up- and down-regulated genes. (G) Reactome pathway enrichment of up- and down-regulated genes. (H) Gene Set Enrichment Analysis (GSEA) of significantly altered gene sets. (I) Protein–protein interaction (PPI) network of significantly altered genes.

RNA sequencing was performed to assess whether oral KerZJ alters the intestinal transcriptome in mice. Principal component analysis (PCA) revealed clear separation between KerZJ-treated and control intestinal samples, indicating distinct transcriptomic profiles (Fig. 3A). A volcano plot summarized the differentially expressed genes (DEGs) in the KerZJ group (Fig. 3B). Relative to controls, 107 genes were upregulated and 87 were downregulated following KerZJ administration (Fig. 3C). A heatmap visualized the expression patterns of DEGs across samples and their relative expression levels (Fig. 3D).

To elucidate the effects of kerZJ on intestinal homeostasis, we performed functional enrichment analysis of DEGs. Gene Ontology (GO) analysis of genes up-regulated in the mouse intestine revealed a pronounced immunological signature, characterized by augmented humoral immunity (B cell activation, immunoglobulin production, complement activation) and potentiated innate immune functions (phagocytic recognition and uptake), together with an enhanced response to bacterial stimuli (Fig. 3E). GO analysis of down-regulated genes demonstrated marked suppression of metabolic and detoxification programs, particularly glutathione/redox and xenobiotic metabolism, accompanied by broader reductions in cellular/chemical homeostasis and circadian processes (Fig. 3E). KEGG pathway analysis was performed to further define the functional consequences of the transcriptional changes induced by kerZJ. For the up-regulated genes, this analysis revealed a trend toward the activation of stress-response and tissue-repair pathways, prominently involving FoxO, Relaxin, and TGF-β signaling (Fig. 3F). While these enrichments did not reach statistical significance after multiple-testing correction, they suggest that kerZJ promotes a transcriptional program conducive to cytoprotection and extracellular matrix remodeling. Conversely, analysis of the down-regulated gene set pointed to the suppression of pathways related to xenobiotic and drug metabolism (notably those involving cytochrome P450 systems), retinol metabolism, and fatty acid degradation (Fig. 3F). This transcriptional signature is consistent with a state of reduced detoxification capacity and attenuated lipid metabolism, potentially contributing to a lowered intestinal metabolic load and enhanced metabolic efficiency.

Corroborating the findings from the GO analysis, Reactome pathway analysis of genes up-regulated by kerZJ in the mouse intestine demonstrated a highly significant and coherent immunological activation. This transcriptional program was characterized by the potentiation of humoral immunity and the enhancement of antigen processing and presentation machinery (Fig. 3G). In parallel, analysis of the down-regulated gene set revealed a coherent suppression of pathways related to xenobiotic and drug metabolism (notably cytochrome P450 and glutathione systems), lipid and sterol catabolism, and retinoid metabolism, alongside broader reductions in intermediary metabolic processes (Fig. 3G). Gene Set Enrichment Analysis (GSEA) demonstrated significant positive enrichment of the CHR6C1 gene set in murine intestinal tissue following kerZJ treatment (Fig. 3H), indicating coordinated up-regulation of genes within the 6C1 cytoband and suggesting region-specific transcriptional regulation. Protein-protein interaction (PPI) network analysis showed that kerZJ induces coordinated up‑regulation of immune and epithelial defense/repair modules alongside coherent down‑regulation of xenobiotic/drug metabolism, glutathione‑dependent detoxification, and fatty‑acid/retinoid catabolism (Fig. 3I). Collectively, these transcriptomic findings indicate that kerZJ shifts the intestinal transcriptome toward heightened immunological preparedness and epithelial protection while reducing metabolic and biotransformation throughput, potentially lowering oxidative burden and stress state.

### Effects of kerZJ on the intestinal proteome in mice

**Fig. 4.**
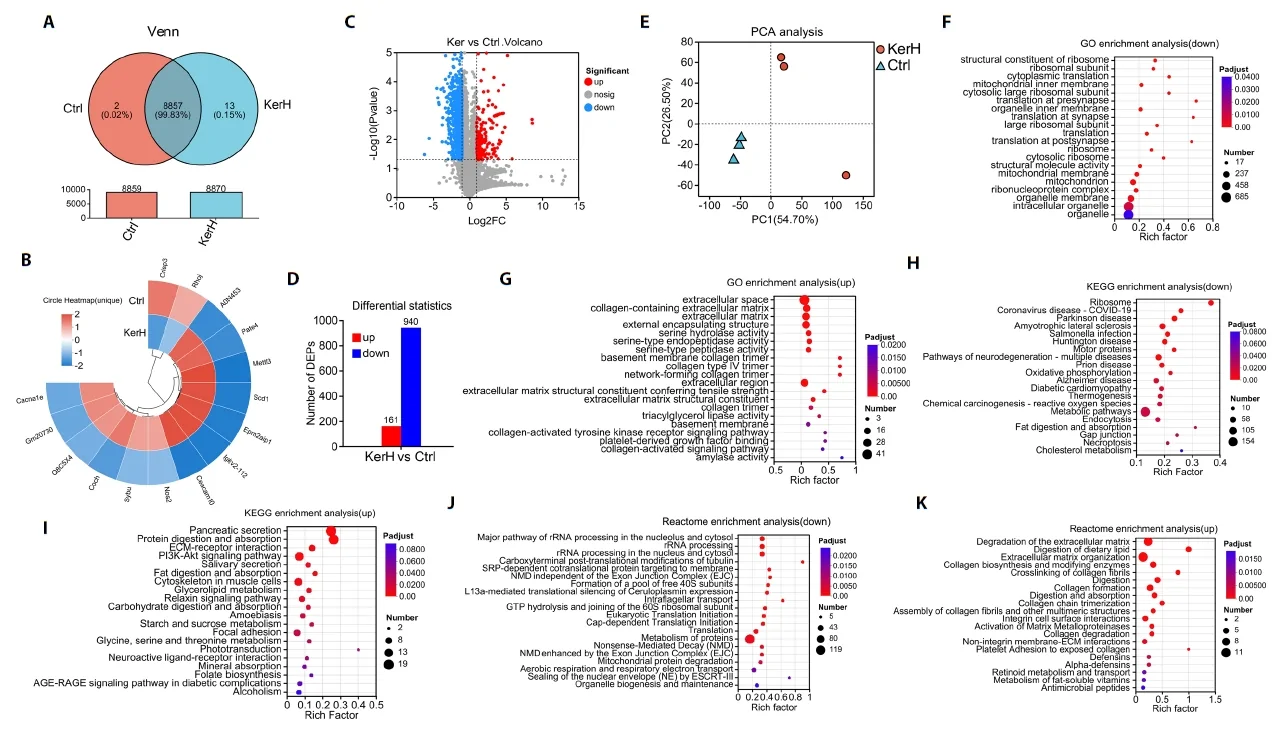
Proteomic analysis and functional pathway annotation in mice. (A) Venn diagram comparing the number of proteins identified between the two groups. (B) Circular heatmap illustrating proteins uniquely identified in each group. (C) Volcano plot highlighting significantly differentially expressed proteins (DEPs). (D) Bar chart quantifying the number of up- and down-regulated DEPs. (E) Principal Component Analysis (PCA) of proteomic profiles across the samples. (F–K) Functional enrichment analysis of DEPs using the (F, G) Gene Ontology (GO), (H, I) Kyoto Encyclopedia of Genes and Genomes (KEGG), and (J, K) Reactome pathway databases.

Proteomic analysis identified 8,859 proteins in the control (Ctrl) group and 8,879 in the high-dose keratinase (KerH) group, indicating that kerZJ treatment did not substantially alter the total number of detectable intestinal proteins (Fig. 4A). Two proteins were uniquely detected in the Ctrl group and 13 in the KerH group; these group-specific proteins are displayed in the circular heatmap (Fig. 4B). Analysis of protein abundance revealed a total of 1,101 differentially expressed proteins (DEPs), visualized in a volcano plot (Fig. 4C). Compared to the Ctrl group, 161 proteins were significantly up-regulated and 940 were significantly down-regulated in the KerH group (Fig. 4D). PCA analysis demonstrated a distinct separation between the proteomic profiles of the two groups, confirming that kerZJ treatment induced a substantial shift in the intestinal proteome (Fig. 4E).

To further elucidate the impact of kerZJ on intestinal homeostasis, we conducted functional enrichment analyses of DEPs. GO enrichment of downregulated proteins was dominated by terms related to ribosomal translation and mitochondrial/organelle membrane components, whereas upregulated proteins were enriched for extracellular matrix (ECM) organization, including collagen IV and basement membrane constituents, alongside strengthened cell-matrix adhesion, ECM-receptor signaling, and increased proteolytic and digestive enzyme activities (Fig. 4F and 4G). These profiles indicate reinforced barrier architecture and enhanced luminal nutrient processing in the intestine of mice treated with kerZJ.

Further analysis with KEGG and Reactome databases corroborated and extended these findings (Fig. 4H–4K). Both databases confirmed that downregulated DEPs converged on pathways suppressing ribosomal translation, oxidative metabolism, and mitochondrial respiration, indicating a calibrated reduction in the cell’s biosynthetic and energetic load. In contrast, upregulated DEPs were consistently enriched in pathways that promote intestinal resilience and efficiency. These included digestive secretion, nutrient absorption, ECM-integrin signaling, and collagen maturation. Notably, Reactome analysis also highlighted the upregulation of antimicrobial peptide defenses, pointing to an enhanced state of innate immunity. Collectively, these results depict a coordinated proteomic shift that fortifies the intestinal barrier and optimizes nutrient handling while alleviating cellular metabolic stress.

### Effects of kerZJ on the intestinal microbiome in mice

**Fig. 5.**
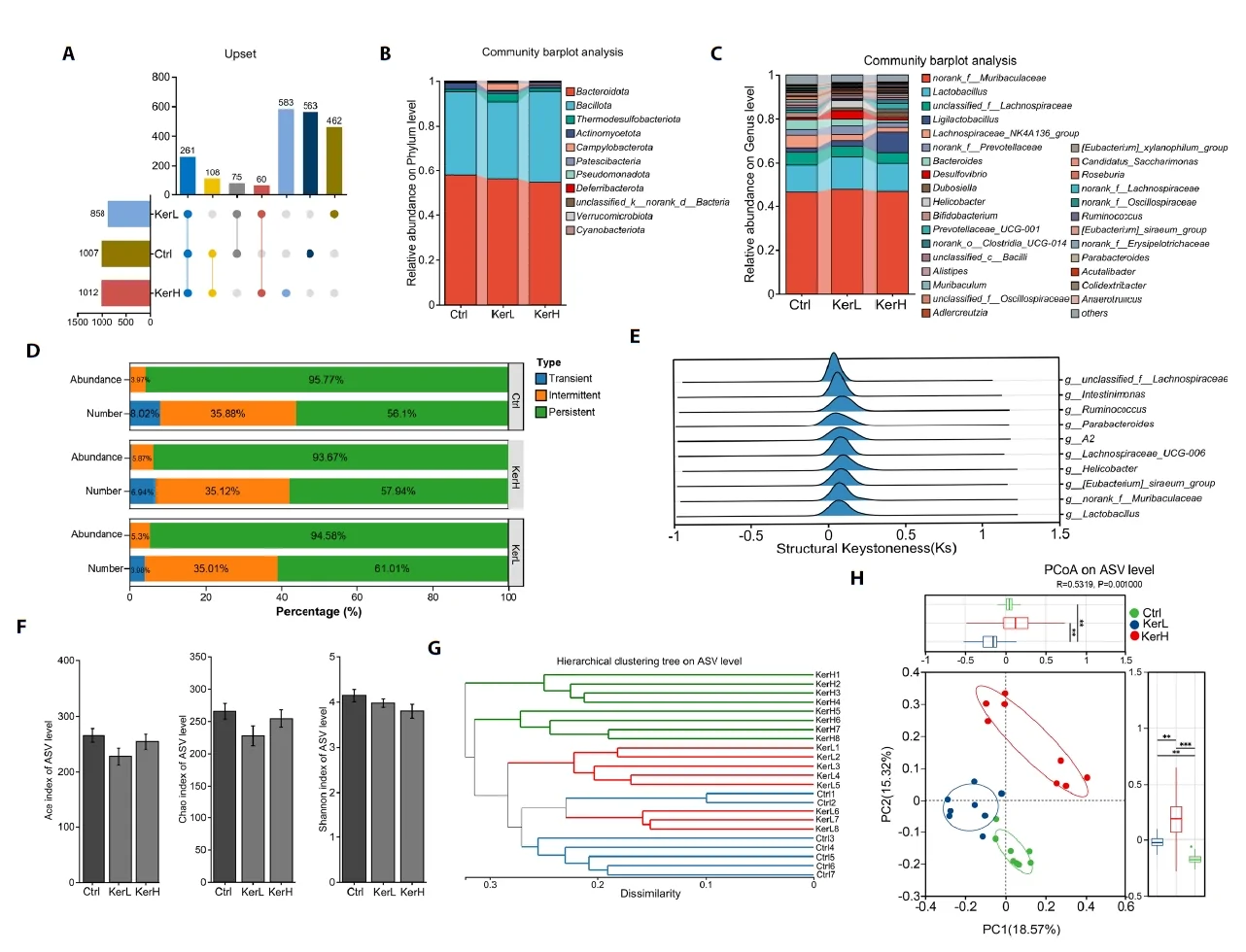
Composition and diversity of the gut microbiota in mice. (A) Venn diagrams showing the number of identified ASVs in each group. (B) Bar chart of relative abundances at the phylum level. (C) Bar chart of relative abundances at the genus level. (D) Bar chart indicating community stability of the gut microbiota in each group. (E) Keystone species peak distribution map quantifying each taxon’s topological importance to community structure. (F) Alpha diversity across the three groups, assessed by ACE, Chao, and Shannon indices. (G) Hierarchical clustering dendrogram based on Bray–Curtis distances for each sample. (H) Principal Component Analysis (PCA) of gut microbiota profiles across all samples. Data are presented as Mean ± standard deviation (SD).

The gut microbiota composition of mice in the Ctrl, KerL, and KerH groups was profiled using high-throughput sequencing of 16S rRNA gene amplicons. A total of 1007, 858, and 1012 Amplicon Sequence Variants (ASVs) were identified in the Ctrl, KerL, and KerH groups, respectively. Among these, 563, 462, and 583 ASVs were unique to each respective group (Fig. 5A). Taxonomic analysis revealed that the microbiota at the phylum level was predominantly composed of *Bacteroidota*, *Firmicutes*, *Thermodesulfobacteriota*, *Actinomycetota*, and *Campylobacterota* across all groups (Fig. 5B). At the genus level, the five most abundant taxa included *norank_f__Muribaculaceae*, *Lactobacillus*, *unclassified_f__Lachnospiraceae*, *Ligilactobacillus*, and *Lachnospiraceae*_NK4A136_group (Fig. 5C). To assess community stability, ASVs were categorized as persistent, intermediate, or transient based on their prevalence and abundance. The relative proportions of these ecological groups were found to be comparable across all groups (Fig. 5D). To identify genera with a disproportionate influence on community architecture, a keystone species analysis was performed. This analysis identified several primary structural keystones of the gut community, including *Lactobacillus*, *norank_f__Muribaculaceae*, various *Lachnospiraceae*-affiliated genera, *Ruminococcus*, *Intestinimonas*, and the *[Eubacterium]_siraeum*_group (Fig. 5E).

The effects of kerZJ on gut microbiota diversity were evaluated using both alpha- and beta-diversity metrics. As shown in Fig. 5F, alpha-diversity indices (ACE, Chao, and Shannon) did not differ significantly among the Ctrl, KerL, and KerH groups, indicating that kerZJ treatment had minimal impact on overall richness and evenness of the mice gut microbiota. Beta-diversity was assessed using hierarchical clustering and principal coordinates analysis (PCoA) (Fig. 5G and 5H). In the hierarchical clustering tree (Fig. 5G), samples from the KerL group clustered together with those from the Ctrl group, whereas KerH samples formed a distinct cluster on separate branches. Consistent with this, the PCoA plot showed that KerL samples clustered tightly and were positioned proximal to Ctrl samples, while KerH samples were clearly separated from both Ctrl and KerL along the principal axes (Fig. 5H). These results indicate that high-dose kerZJ (KerH), but not low-dose kerZJ (KerL), induces a marked shift in overall community composition relative to controls, without altering alpha-diversity.

**Fig. 6.**
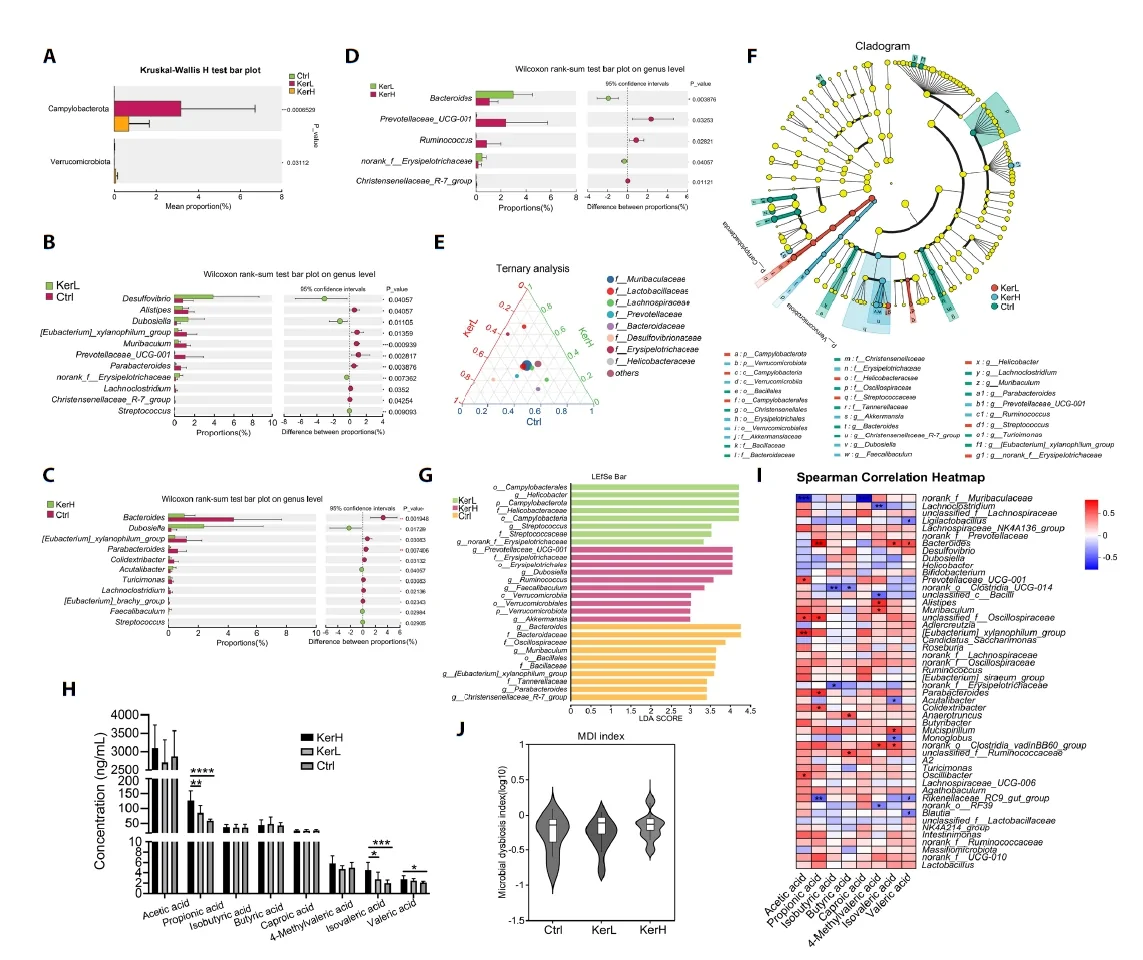
Alterations in the gut microbial community across the three groups. (A) Bar plot showing significantly altered bacterial phyla among the groups. (B–D) Bar plots showing significantly altered bacterial genera in each pairwise group comparison. (E) Ternary plot depicting the distribution and relative abundances of major bacterial taxa across groups. (F) LEfSe analysis of intestinal microbiota across the three groups. (G) Linear discriminant analysis (LDA) scores highlighting differentially abundant taxa. Histogram shows taxa enriched in each group. (H) Concentrations of short-chain fatty acids (SCFAs) in each group. (I) Spearman correlation heatmap illustrating associations between specific bacterial taxa and SCFAs. (J) Gut microbial dysbiosis index calculated from community composition, using the Ctrl group as the reference. Values are expressed as Means ± standard deviation (SD). (^*^p < 0.05, ^**^p < 0.01, ^***^p < 0.005, ^****^p < 0.001)

To identify kerZJ-induced alterations in gut microbiota composition, differential abundance analyses were conducted at multiple taxonomic levels. At the phylum level, the relative abundances of *Campylobacterota* and *Verrucomicrobiota* were significantly elevated in the KerH group compared with controls (Fig. 6A). At the genus level, kerZJ administration significantly increased the abundances of *Desulfovibrio, norank_f__Erysipelotrichaceae*, *Streptococcus*, *Acutalibacter*, and *Faecalibaculum* relative to the Ctrl group, while decreasing those of *Alistipes, [Eubacterium]_xylanophilum*_group, *Muribaculum*, *Prevotellaceae*_UCG-001, *Parabacteroides*, *Lachnoclostridium*, *Christensenellaceae*_R-7_group, *Bacteroides*, *Turicimonas*, and *[Eubacterium]_brachy*_group (Fig. 6B and 6C). In direct comparison with the low-dose group (KerL), KerH showed significantly lower relative abundances of *Bacteroides* and *norank_f_Erysipelotrichaceae*, alongside significant increases in *Prevotellaceae*_UCG-001, *Ruminococcus*, and *Christensenellaceae*_R-7_group (Fig. 6D). Ternary analysis visualized group-wise compositional biases among dominant genera and indicated that the family *Lactobacillaceae* was more enriched in both kerZJ-treated groups than in controls (Fig. 6E). Linear Discriminant Analysis Effect Size (LEfSe) further identified differentially abundant taxa (Fig. 6F), with 10, 8, and 10 phylotypes achieving significant Linear Discriminant Analysis (LDA) scores in the Ctrl, KerL, and KerH groups, respectively (Fig. 6G). The genus *Prevotellaceae*_UCG-001 and *Dubosiella*, the family *Erysipelotrichaceae*, and the order *Erysipelotrichales* were significantly enriched (LDA > 4) in the KerH group.

The effects of kerZJ on short-chain fatty acid (SCFA) concentrations were quantified. As shown in Fig. 6H, kerZJ significantly increased several SCFAs, including propionic, isovaleric, and valeric acids, in a dose-dependent manner. Given that SCFAs are primarily generated by microbial fermentation of dietary fiber, we constructed a Spearman correlation heatmap to assess associations between specific bacterial genera and SCFA levels (Fig. 6I). This revealed that several genera, including *Lachnospiraceae*_NK4A136_group, *norank_f_Oscillospiraceae*, and *Mucispirillum*, were positively correlated with all measured SCFAs (Fig. 6I). Importantly, although kerZJ administration altered gut microbial composition, this did not translate to significant dysbiosis, as reflected by a microbial dysbiosis index that was not significantly different from the control group (Fig. 6J).

## Discussion

Keratinases represent a critical class of proteases with expanding industrial and biomedical applications. Integration of artificial intelligence and machine learning into the enzyme engineering toolkit is accelerating the development of bespoke keratinase variants with optimized properties for specific industrial needs (Pei et al., 2025; Su et al., 2020). One of the most promising applications is their use as feed additives to enhance growth performance and nutrient digestibility in poultry (Saeed et al., 2024; Wang et al., 2011). However, a critical knowledge gap persists regarding the physiological toxicity and long-term safety of keratinase consumption, particularly their impact on the host's gut ecosystem. The present study provides the first comprehensive multi-omic assessment of keratinase kerZJ’s impact on mice gut homeostasis, demonstrating its safety profile and revealing unexpected beneficial effects on intestinal barrier function, immune modulation, and metabolic homeostasis.

Our results demonstrate that kerZJ administration is well-tolerated and does not induce systemic toxicity in mice (Fig. 1). The kerZJ strengthens intestinal barrier integrity through multiple complementary mechanisms. Histological analysis revealed preserved intestinal architecture with no pathological changes, and immunofluorescence confirmed maintained expression of critical tight junction proteins (ZO-1, claudin-1, occludin-1) (Fig. 2). Proteomic analysis revealed significant upregulation of extracellular matrix (ECM) components, particularly collagen IV and basement membrane constituents, alongside enhanced ECM-integrin signaling pathways (Fig. 4). This coordinated upregulation of structural proteins suggests that kerZJ promotes active barrier reinforcement rather than merely maintaining baseline integrity (Karamanos et al., 2021; Liu et al., 2022). The enhanced ECM organization likely provides additional mechanical support to the epithelium and may facilitate improved nutrient absorption through optimized epithelial-stromal interactions (Karamanos et al., 2021). These findings align with the transcriptomic data showing activation of TGF-β and Relaxin signaling pathways, both known regulators of ECM remodeling and tissue repair (Fig. 3). Considering that no abnormal inflammatory markers or epithelial damage indicators were detected, these results may demonstrate that these structural enhancements represent physiological adaptation rather than compensatory responses to injury.

KerZJ induced an immunological reprogramming characterized by enhanced preparedness without pathological activation. Transcriptomic analysis revealed coordinated upregulation of humoral immunity components, including B cell activation, immunoglobulin production, and complement activation, alongside potentiated innate immune functions such as phagocytic recognition and bacterial response mechanisms (Fig. 3). Proteomic data corroborated these findings, demonstrating increased antimicrobial peptide defenses and enhanced antigen processing machinery (Fig. 4). This balanced immune enhancement appears to prime the intestinal immune system for rapid response to potential threats while avoiding the inflammatory sequelae typically associated with immune activation. The maintained levels of sIgA and absence of systemic inflammatory markers indicate that kerZJ promotes immunological vigilance rather than active inflammation (Fig. 1C). This immunomodulatory profile is particularly valuable for feed applications, as it may enhance disease resistance (Mohai Ud Din et al., 2025). Nattokinase, a well-established thrombolytic enzyme, has been shown to exert significant anti-inflammatory effects, preserve epithelial barrier integrity, and remodel the gut microbiota toward a SCFA-productive state in a mouse model of colitis (Liang et al., 2022; Zhou et al., 2020). Notably, our prior investigation revealed that kerZJ possesses substantial thrombolytic activity (Peng et al., 2023), and the present study confirmed its robust immunomodulatory, gut barrier-reinforcing, and microbiota-modulating capabilities. These findings position kerZJ as a promising alternative to nattokinase for the development of thrombolytic dietary supplements (Sheng et al., 2023).

KerZJ exhibited profound impact on digestive and metabolic functions. Proteomic analysis revealed significant upregulation of digestive enzymes and nutrient absorption pathways, suggesting enhanced luminal nutrient processing capacity (Fig. 4). This was accompanied by a paradoxical downregulation of ribosomal translation and mitochondrial respiration pathways, indicating a shift toward more efficient cellular metabolism with reduced biosynthetic burden. The coordinated suppression of fatty acid degradation, retinol metabolism, and cytochrome P450 systems suggests a metabolic reprogramming that prioritizes nutrient utilization over catabolism. This metabolic shift was functionally validated by the dose-dependent increase in SCFA production, particularly propionic, isovaleric, and valeric acids (Fig. 6H). The enrichment of *Lactobacillaceae* family members in both treatment groups further supports enhanced fermentative capacity (Montgomery et al., 2024). These metabolic adaptations likely contribute to improved feed efficiency and growth performance observed in previous poultry studies (Odetallah et al., 2005; Saeed et al., 2024), while the increased SCFAs production provides additional benefits including enhanced barrier function, anti-inflammatory effects, and improved energy harvest from dietary substrates (Tan et al., 2014).

KerZJ induced gut microbiota alterations without causing dysbiosis. Based on our results, the alpha diversity of gut microbiota showed no significant differences across all groups, while beta diversity analysis revealed dose-dependent compositional changes primarily at high dose. LEfSe identified *Erysipelotrichaceae* and *Prevotellaceae*_UCG‑001 among the discriminant taxa at high dose. While certain taxa with context-dependent implications (for example, *Desulfovibrio*, Erysipelotrichaceae; phylum-level *Campylobacterota* and *Verrucomicrobiota*) increased, the absence of histopathological abnormalities, preserved tight junctions, stable alpha diversity, and favorable proteome/transcriptome signatures argue against adverse gut effects under the conditions tested. These compositional changes, together with elevated SCFAs and their positive associations with fermentative taxa, support a model whereby kerZJ fosters a metabolically favorable, resilient gut ecosystem (Khasanah et al., 2024; Su et al., 2025).

Collectively, these findings extend prior evidence that keratinases can improve animal performance and underscore kerZJ as a multipurpose protease with translational potential in feed. In vivo, kerZJ preserved gut barrier integrity, modulated mucosal immunity in a protective manner, enhanced nutrient processing, and promoted SCFA-associated microbiota without detectable toxicity. These properties provide a mechanistic basis for deploying kerZJ as a safe, functional enzyme in sustainable animal nutrition and as a candidate for other biotechnological/health applications. Notably, this study was conducted in mice over a relatively short 30-day period and validating the efficacy and safety of kerZJ in poultry and other livestock under practical diet formulations is essential prior its agricultural applications.

## Data Availability

The data that support the findings of this study are available from the corresponding author upon reasonable request.
